# MELK is a novel therapeutic target in high-risk neuroblastoma

**DOI:** 10.18632/oncotarget.23515

**Published:** 2017-12-20

**Authors:** Shan Guan, Jiaxiong Lu, Yanling Zhao, Yang Yu, Hui Li, Zhenghu Chen, Zhongcheng Shi, Haoqian Liang, Mopei Wang, Kevin Guo, Xiangmei Chen, Wenjing Sun, Shayahati Bieerkehazhi, Xin Xu, Surong Sun, Saurabh Agarwal, Jianhua Yang

**Affiliations:** ^1^ Xinjiang Key Laboratory of Biological Resources and Genetic Engineering, College of Life Science and Technology, Xinjiang University, Urumqi 830046, China; ^2^ Texas Children's Cancer Center, Department of Pediatrics, Dan L. Duncan Cancer Center, Baylor College of Medicine, Houston, TX 77030, USA; ^3^ Department of Pathology, University of Texas MD Anderson Cancer Center, Houston, TX 77030, USA; ^4^ Department of Cardiothoracic Surgery, Zhujiang Hospital, Southern Medical University, Guangzhou 510282, China; ^5^ Department of Tumor Chemotherapy and Radiation Sickness, Peking University Third Hospital, Beijing 100083, China; ^6^ Peking University Health Science Center, Beijing 100083, China; ^7^ Laboratory of Medical Genetics, Harbin Medical University, Harbin 150081, China

**Keywords:** neuroblastoma, MYCN/MYC, MELK, chemotherapy, OTSSP167

## Abstract

Maternal embryonic leucine zipper kinase (MELK) is known to modulate intracellular signaling and control cellular processes. However, the role of MELK in oncogenesis is not well defined. In this study, using two microarray datasets of neuroblastoma (NB) patients, we identified that MELK expression is significantly correlated to poor overall survival, unfavorable prognosis, and high-risk status. We found that MELK is a direct transcription target of MYCN and MYC in NB, and MYCN increases MELK expression via direct promoter binding. Interestingly, knockdown of MELK expression significantly reduced the phosphorylation of target protein Retinoblastoma (pRb) and inhibited NB cell growth. Furthermore, pharmacological inhibition of MELK activity by small-molecule inhibitor OTSSP167 significantly inhibited cell proliferation, anchorage-independent colony formation, blocked cell cycle progression, and induced apoptosis in different NB cell lines including a drug-resistant cell line. Additionally, OTSSP167 suppressed NB tumor growth in an orthotopic xenograft mouse model. Overall, our data suggest that MELK is a novel therapeutic target for NB and its inhibitor OTSSP167 is a promising drug for further clinical development.

## INTRODUCTION

Neuroblastoma (NB) is the most common extracranial neoplasm in children and contributes to about 15% of all pediatric cancer-related deaths (1). Despite major advances in therapies over the past decade, the overall outcome for high-risk NB patients is still unacceptable [1]. Current therapies include chemotherapy drugs that are highly toxic to healthy cells and have significant long-term side effects. Therefore, developing novel targeted therapies for high-risk NB is critical to achieve higher efficacy and to alleviate adverse effects. *MYCN* amplification is a strong characteristic of high-risk NB patients and serves as a genetic marker of disease [2, 3]. Finding therapeutic strategies to directly target MYCN is a difficult task due to its protein structure. Thus, identifying and characterizing druggable targets in MYCN regulators and transcriptional targets in NB may help us to develop efficient therapeutic strategies.

Protein kinases play an essential role in the regulation of cell survival and proliferation [4]. Different kinases such as anaplastic lymphoma kinase (ALK) [5], Aurora kinase [6], RET receptor tyrosine kinase [7] have been shown to be potential therapeutic targets in various cancers, including NB [8]. Maternal embryonic leucine zipper kinase (MELK) is a serine/threonine kinase overexpressed in various organ-specific stem cells and cancers [9, 10]. High *MELK* expression predicts a poor prognosis of many cancer types, including but not limited to breast cancer [11], astrocytoma [12] and glioblastoma [13]. MELK has been shown to promote cancer cell survival and tumor cell differentiation [14]. Furthermore, although MELK has been suggested to form a heterotrimeric protein complex with the oncogenic transcription factors c-Jun and FoxM1 in cancer stem cells, MELK lacks the binding ability in normal progenitor and stem cells [15, 16]. Therefore, inhibition of the kinase activity of MELK may disrupt MELK-mediated malignancy in cancer cells, while having a minimal effect on normal cells [13].

The molecular mechanism regulating MELK overexpression in cancer cells and the role of MELK in NB tumorigenesis remains ambiguous. In this study, we analyzed a large cohort of NB patients and identified that higher MELK expression is correlated with poor overall survival, prognosis, and overall outcome. Moreover, MELK expression is higher in tumors from high-risk NB patients. Using ChIP-qPCR assays, we showed that MYCN directly binds at the E-box binding motifs present at the *MELK* gene promoter and promotes transcription. Additionally, the *MYCN* or *MYC* knockdown led to decreased MELK mRNA and protein levels in NB cells, whereas *MYCN* or *MYC* overexpression led to increased mRNA and protein levels of MELK in NB cells. Furthermore, MELK small molecule inhibitor OTSSP167 inhibited NB growth both *in vitro* and *in vivo* by inducing apoptosis. This is the first report that shows the oncogenic role and regulation of MELK in NB. Novel MELK inhibitor OTSSP167 is shown to reduce growth of various cancer types [17]. The orally active compound OTSSP167 is currently in phase-I clinical trials for solid tumors (ClinicalTrails.gov # NCT01910545) and is fast approaching for further clinical development. Our pre-clinical data suggest that MELK is an attractive therapeutic target in *MYCN- and MYC*-driven cancers, such as NB.

## RESULTS

### *MELK* expression is a prognostic marker for high-risk NB

**Figure 1:**
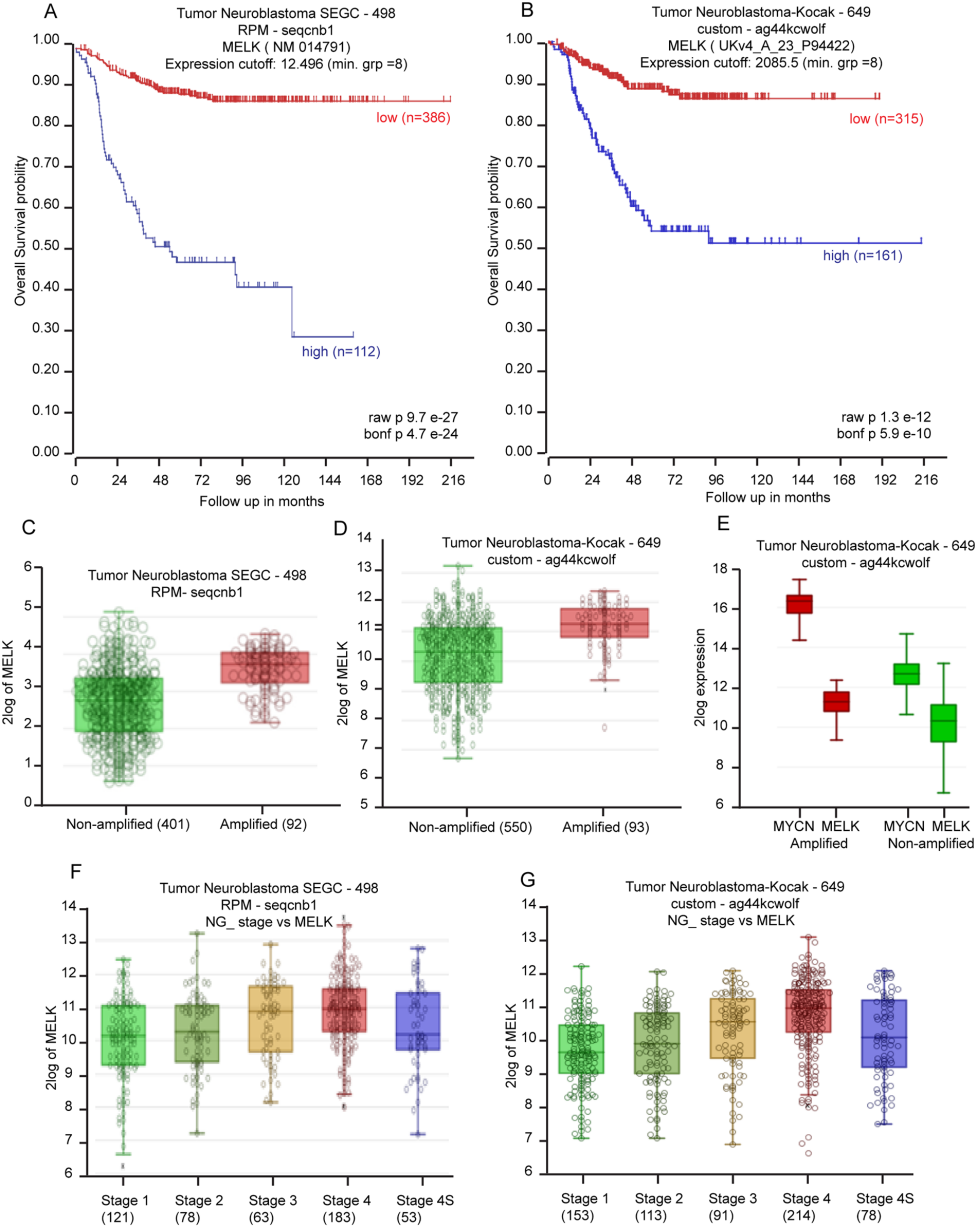
*MELK* is a prognostic indicator in NB **(A** and **B)** Kaplan-Meier curves show the probability of overall survival based on MELK expression level of 498 patients in the SEGC dataset (A) and 649 patients in the Kocak dataset (B). **(C** and **D)** R2-tumor neuroblastoma SEGC dataset (C) and Kocak dataset (D) show higher *MELK* expression level in MYCN-amplified cases in comparison to MYCN-non-amplified cases. **(E)** The Kocak dataset shows significant correlations of *MELK* with *MYCN* expression levels. **(F** and **G)** R2-tumor neuroblastoma SEGC dataset (F) and Kocak dataset (G) show correlation of *MELK* expression level with NB disease stages.

To evaluate how transcription of *MELK* correlates with NB outcomes, we analyzed large clinical cohorts of NB patients using the R2-data analysis platform. Kaplan-Meier analyses of datasets revealed that low *MELK* transcript levels strongly correlated with better overall and event-free survival for the Tumor Neuroblastoma SEGC dataset (n = 498) (p < 9.7E-27) and Kocak dataset (n = 649) (p < 1.3e-12) (Figure 1A, 1B). Interestingly, we observed higher *MELK* expression levels (p < 0.001) in *MYCN*-amplified NB tumors compared to those in *MYCN*-nonamplified ones (Figure 1C, 1D). There were significant corrrelations of *MELK* with *MYCN* expression levels according to Kocak dataset (Figure 1E). In addition, more aggressive, higher stage tumors had significantly higher *MELK* expression, suggesting that *MELK* has a role in de-differentiated invasive malignancy (Figure 1F, 1G). These findings indicate that *MELK* expression is an important factor in the biology and therapeutic response for high-risk NB.

### MYCN and MYC promotes *MELK* expression in NB

**Figure 2:**
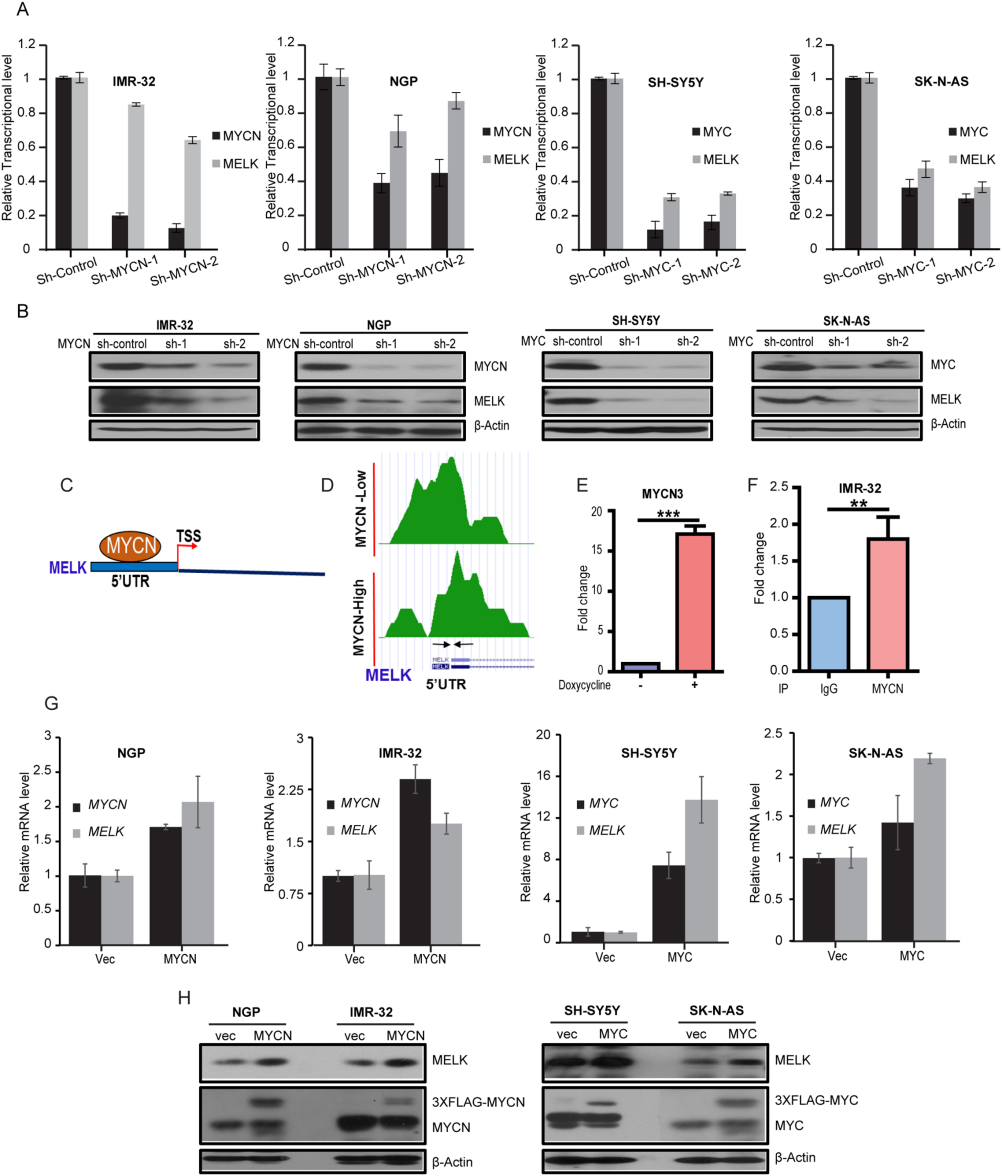
MELK is a transcription target of MYCN/MYC in NB **(A)** Stable sh-RNA-mediated knockdown of *MYCN/MYC*. The qPCR analyses of *MYCN/MYC* and *MELK* expression levels were shown. **(B)** Total proteins of MYCN/MYC and MELK were measured in the indicated cells. **(C** and **D)** MYCN ChIP-seq analysis showed binding peaks of MYCN on *MELK* 5’UTR. TSS- Translation Start Site. Designed primers for ChIP-seq analysis is shown with arrows under the peaks. These primers encompass the E-Box motif. **(E)** ChIP-qPCR analysis for MYCN binds on *MELK* 5’UTR site in MYCN3 cell line. Fold change with or without doxycycline treatment on MYCN3 cell line was shown. **(F)** ChIP-qPCR analysis for MYCN binds on *MELK* 5’UTR site in IMR-32 NB cell line. IP-IgG and IP-MYCN of MELK-ChIP assay on IMR-32 cells were shown. **(G)** qPCR analysis of *MYCN/MYC* and *MELK* mRNA levels in NB cell lines with stable *MYCN/MYC*-overexpression were shown. **(H)** MYCN/MYC and MELK protein levels in NB cell lines with stable *MYCN/MYC*-overexpression were shown.

To evaluate the correlation between MELK and MYCN/MYC expression in NB cells, we performed an sh-RNA mediated knockdown of *MYCN* in two *MYCN*-amplified NB cell lines, NGP and IMR-32, as well as knockdown of *MYC* in two *MYCN*-non-amplified NB cell lines, SK-N-AS and SH-SY5Y. Results showed that stable knockdown of *MYCN* or *MYC* led to both reduced MELK mRNA and protein in NB cell lines tested (Figure 2A, 2B). These results indicated that *MELK* is a MYCN/MYC transcriptional target in NB. Furthermore, analysis of published ChIP-sequencing dataset in NB [18] revealed that MYCN directly binds to the E-Box motif present at the *MELK* 5’UTR (Figure 2C, 2D). To verify these results, we performed individual ChIP-qPCR assays in a MYCN inducible cell line (MYCN3), where MYCN levels can be controlled with doxycycline treatments [18]. With doxyxcline treatment, MYCN binding increased about 17 fold in comparison to the non-treated control (Figure 2E). Similarly, in a ChIP-qPCR assay with IMR-32 cells, anti-MYCN antibody pulled down about a 2 fold more *MELK* 5’UTR genomic sequence in comparison to the control IgG (Figure 2F). To further confirm the correlation between MELK and MYCN/MYC expressions in NB, we overexpressed MYCN with a 3XFLAG tag in *MYCN*-amplified IMR-32, NGP cell lines, and MYC with a 3XFLAG tag in *MYCN*-non-amplified SK-N-AS, SH-SY5Y NB cell lines. Indeed, our results demonstrated that further stable overexpression of *MYCN* or *MYC* greatly increased both mRNA and protein levels of MELK in these four NB cell lines tested (Figure 2G, 2H). These data further support that *MELK* is a MYCN/MYC transcriptional target.

### MELK plays oncogenic role in NB

**Figure 3:**
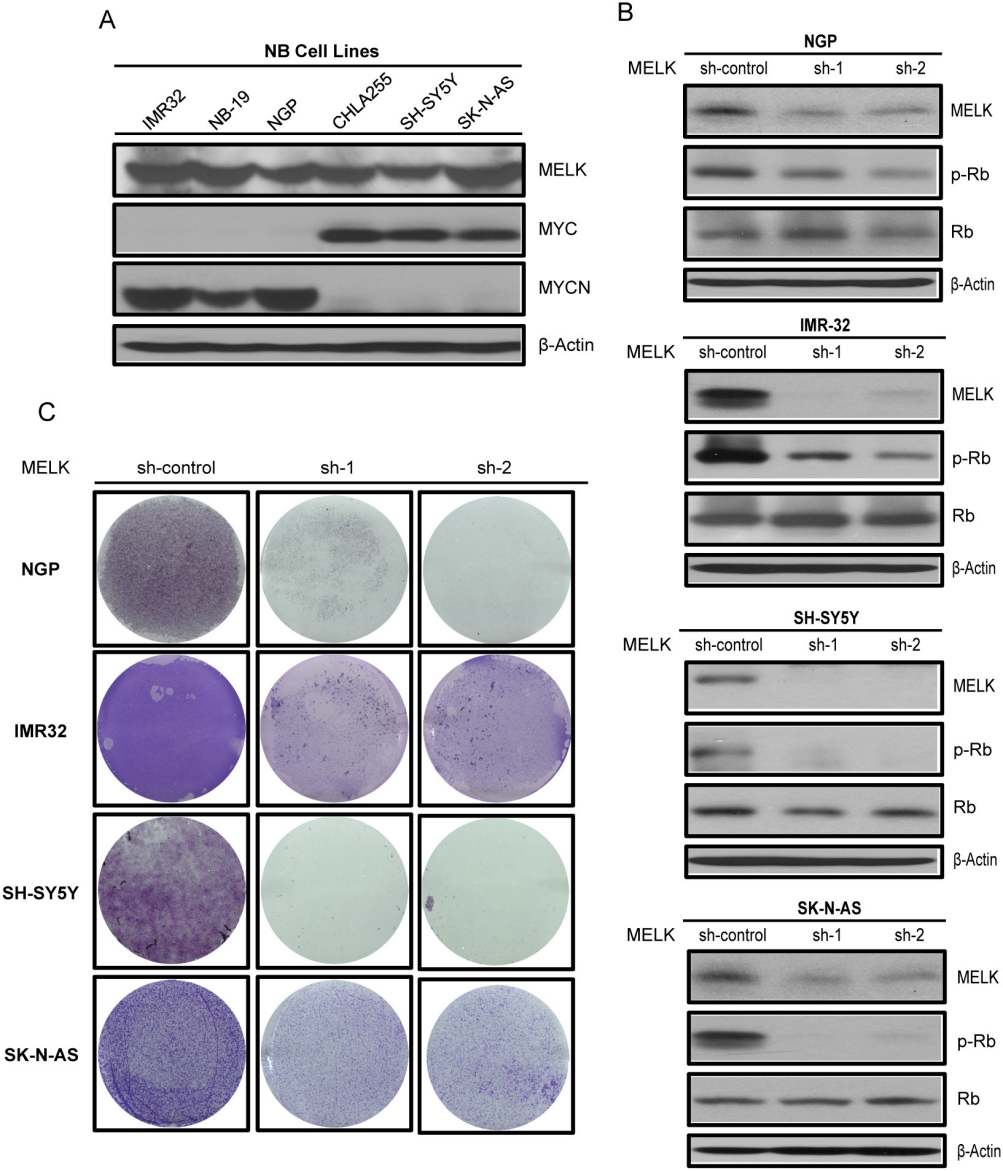
MELK plays an oncogenic role in NB **(A)** MELK protein levels were examined by immunoblotting assay in NB cell lines listed. **(B)** Effects of *MELK* knockdown on p-Rb levels in NB cell lines were shown by immunoblotting assay. **(C)** The effect of MELK knockdown on NB colony formation potential were shown.

To further understand the role of MELK in NB, we first examined the overall level of MELK protein in a panel of six NB cell lines consisting of three *MYCN*-amplified, IMR-32, NB-19 and NGP, and three *MYCN*-non-amplified, CHLA-255, SH-SY5Y and SK-N-AS cell lines (Figure 3A). Results showed abundant levels of MELK in all the cell lines tested, and that MYCN/MYC increases the expression of this kinase protein. Next, we performed an sh-RNA mediated knockdown of *MELK* in four NB cell lines (IMR-32, NGP, SK-N-AS and SH-SY5Y). As expected, the stable knockdown of *MELK* reduced MELK protein levels with both of the sh-RNA knockdown constructs used (Figure 3B). *MELK* knockdown also significantly inhibit the anchorage-independent colony formation and growth in all NB cell lines tested (Figure 3C). These results suggest that MELK contributes to cellular proliferation and is necessary for NB cell survival. MELK kinase regulates cell cycle progression via phosphorylating and activating the tumor suppressor Retinoblastoma (pRb) protein [19]. MYCN is also shown to regulate and inactivate Rb proteins in NB [20, 21]. Consistantly, our results showed that MELK knockdown significantly inhibit the Rb phosphorylation in all the NB cell lines tested (Figure 3B). These results confirm that MELK plays a oncogenic role via regulating the activation of Rb protein in NB.

### MELK inhibitor OTSSP167 potently suppresses NB growth *in vitro*

**Figure 4:**
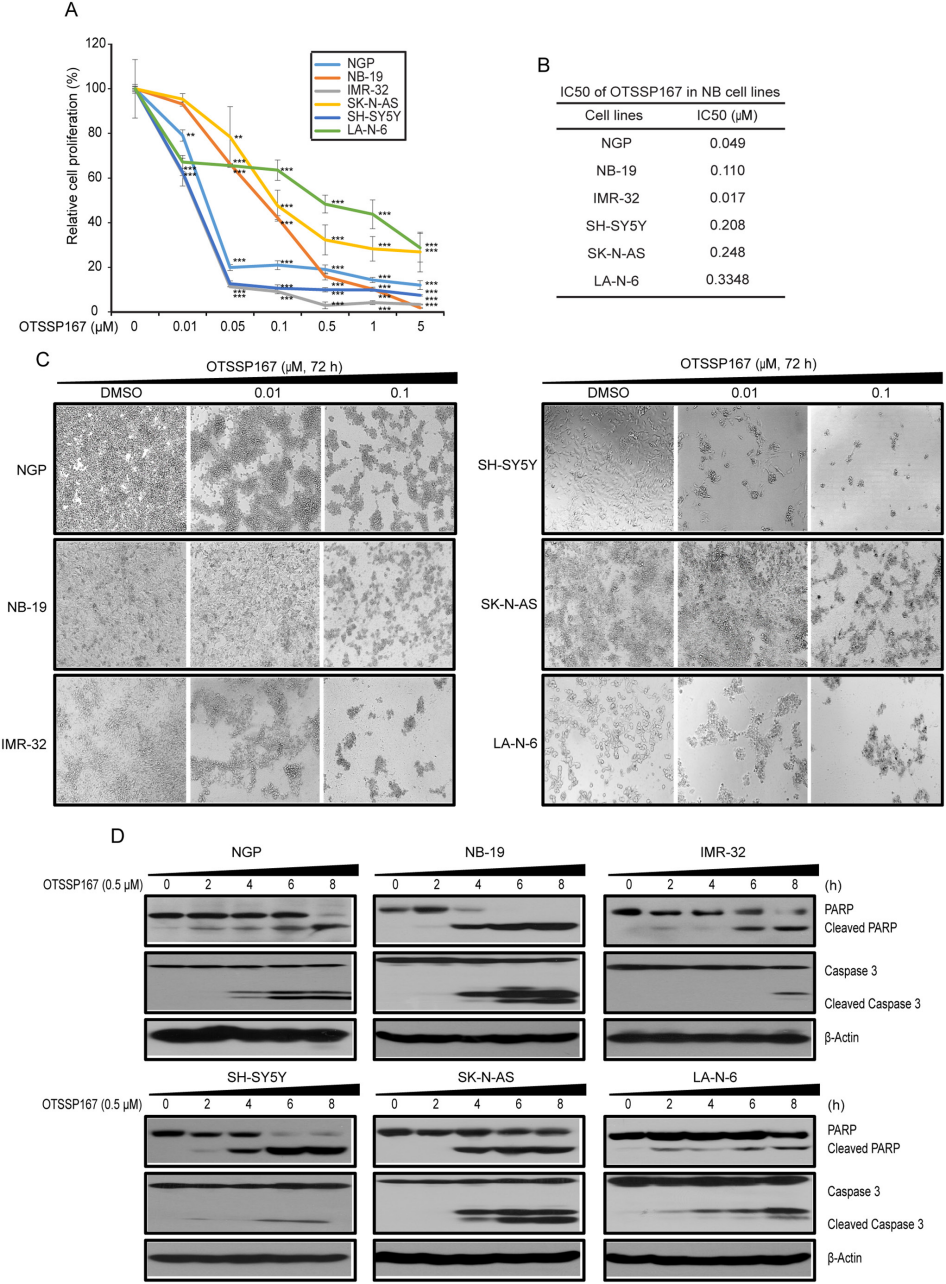
MELK inhibitor OTSSP167 inhibits cell proliferation and induces apoptosis in NB **(A)** The effects of OTSSP167 on the proliferation of a panel of six NB cell lines using cck-8 assay were shown. **(B)** IC50 calculation of cell lines showen in (A) by using PRISM 5.0. **(C)** Effects of OTSSP167 on NB cell morphology were shown. **(D)** Effects of OTSSP167 on PARP and Caspase-3 cleavages in NB cells were shown by immunoblotting assay.

**Figure 5:**
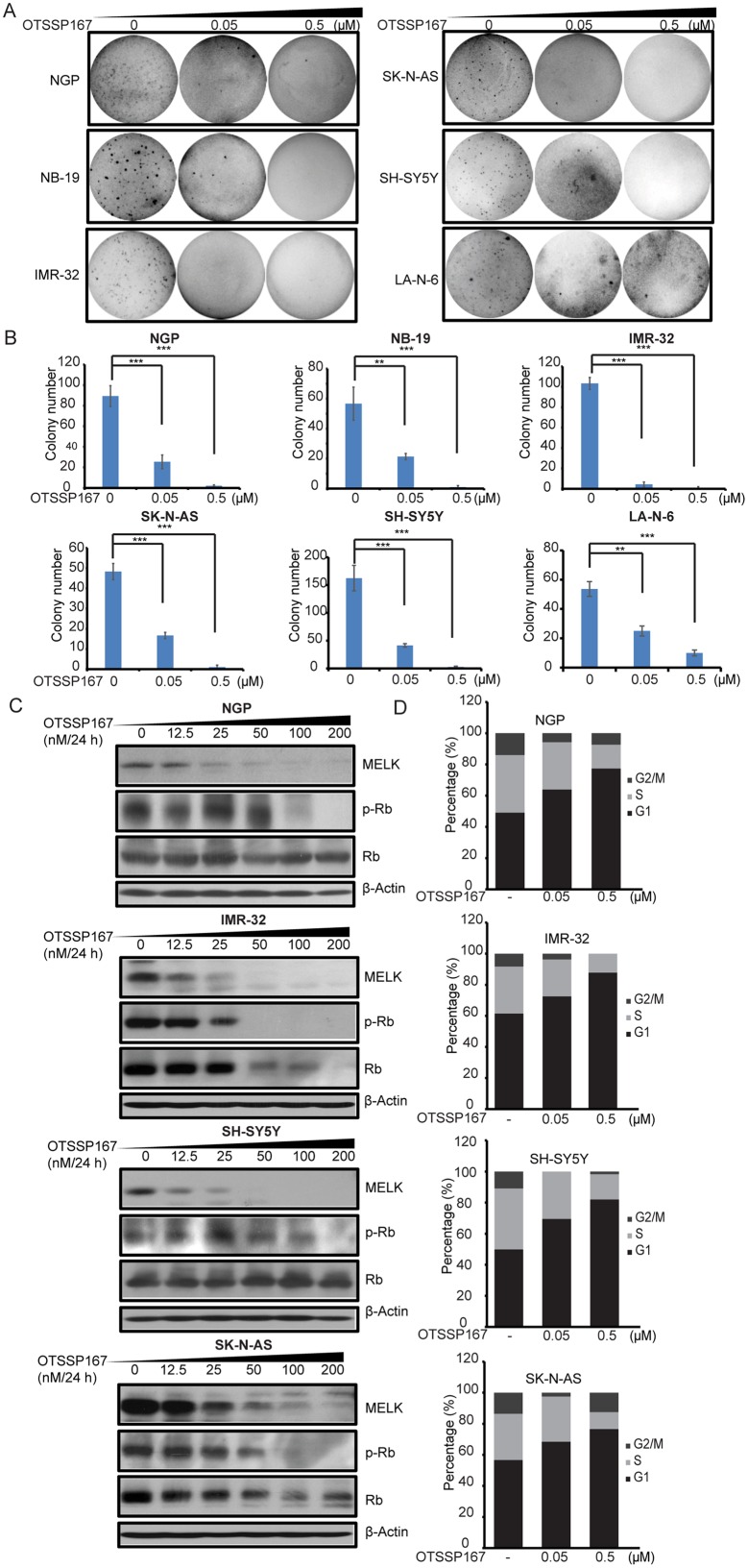
MELK inhibitor OTSSP167 exhibits inhibitory effects in NB **(A)** The effects of OTSSP167 on anchorage-independent colony formation and growth in soft agar assay were shown. **(B)** Number counts of (A). **(C)** The effects of OTSSP167 on p-Rb level in NB cells were shown by immunoblotting assay. The anti-β-actin antibody was used as a loading control for whole cell extracts. **(D)** The effects of OTSSP167 on NB cell cycle by flow cytometry analysis.

Our data suggest that MELK inhibition is a potential therapeutic strategy for high-risk NB. We evaluated therapeutic efficacy of a novel small-molecule MELK inhibitor, OTSSP167 [22, 23], in NB cell lines. OTSSP167 significantly inhibit NB proliferation and cellular growth at low nM doses and in a dose-dependent manner, with relatively low IC_50_ values ranging from most sensitive in IMR-32 (17 nM) to less sensitive in a drug-resiistant NB cell line LA-N-6 (334.8 nM) (Figure 4A, 4B, 4C). Along with reduced growth, cell morphological changes were observed in OTSSP167 treated NB cell lines (Figure 4C). In addition, OTSSP167 induces apoptosis in NB cells, as demonstrated by the PARP and Caspase-3 cleavage assays (Figure 4D). Furthermore, OTSSP167 treatments significantly inhibit anchorage-independent colony formation in a panel of NB cell lines tested (Figure 5A, 5B). This soft-agar colony formnation assay showed that OTSSP167 mediated MELK inhibition significantly inhibit NB growth *in vitro*. According to prior studies and our data shown above, a depletion of MELK activates the Rb pathway and induces cell cycle arrest [19]. To further validate if OTSSP167 mediated MELK inhibibition can inhibit the Rb phosphorylation and block cell cycle progression, we performed immunoblotting and cell cycle assays in four NB cell lines (Figure 5C, 5D). Indeed, the results showed that OTSSP167 treatment significantly inhibit the MELK levels and consequently inhibit the Rb phosphorylation, and block the cell cycle progression at G1 phase, in a dose-dependent manner and in all the NB cell lines tested (Figure 5C, 5D). These data strongly suggest that pharmacological inhibition of MELK inhibits NB proliferation, colony formation, significantly induce apoptosis by blocking the cell cycle progression.

### MELK inhibitor OTSSP167 potently inhibits NB tumor growth *in vivo*

**Figure 6:**
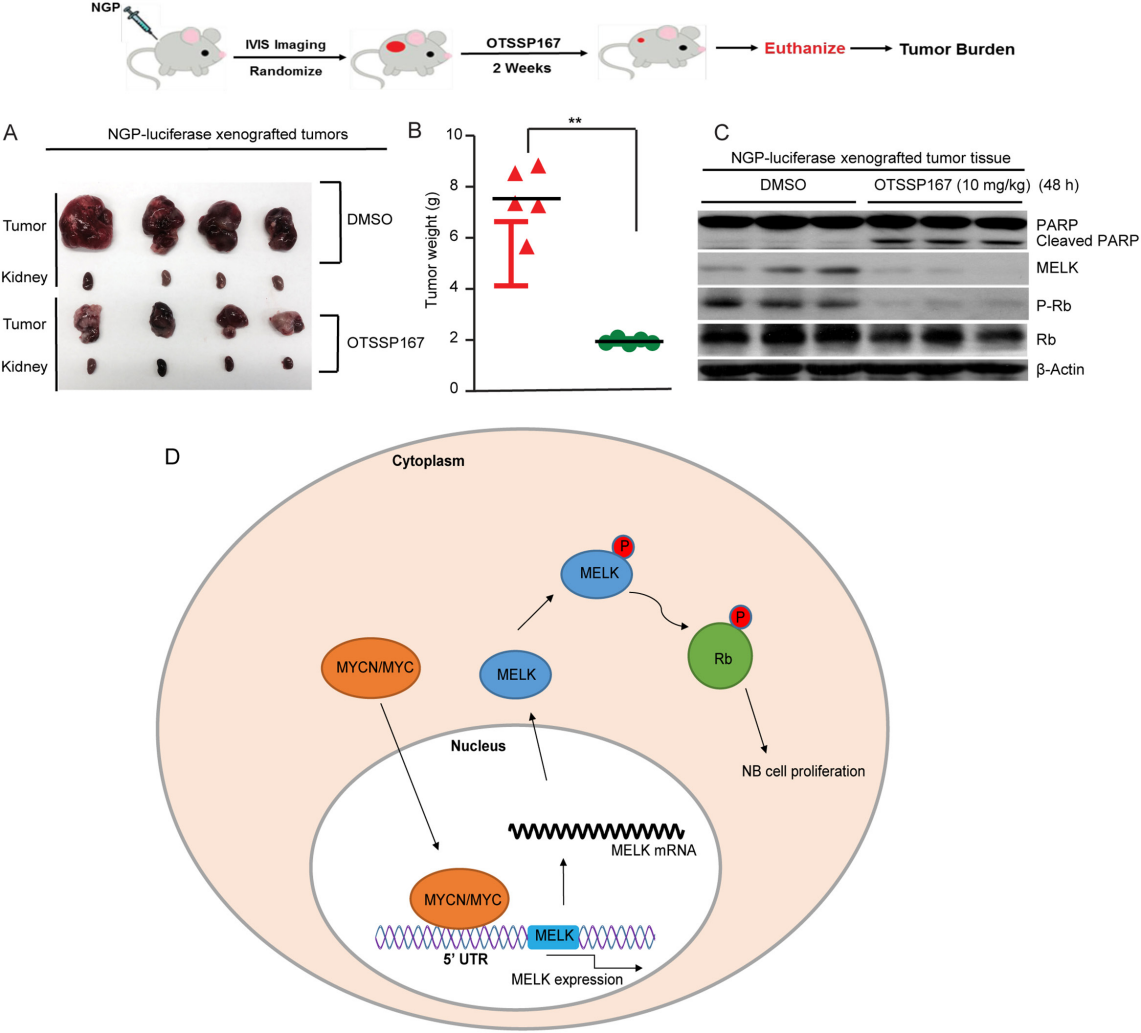
MELK inhibitor OTSSP167 inhibits Nb tumor growth (**A** and **B**) NGP-luciferase xenografts were treated with DMSO or 10 mg/kg of OTSSP167 daily for 14 days. After treatment cycles, tumors and corresponding kidneys were photographed (A) and weighted (B). **(C)** Mice with NB tumors were treated with DMSO or 10 mg/kg of OTSSP167 daily for 48 hours. Effects of OTSSP167 on Rb phosphorylation and apoptotic marker PARP cleavage in the established NB tumor *in vivo* were shown. **(D)** A working model of MELK function in NB cells was shown.

To determine the *in vivo* effects of OTSSP167, we used an orthotopic NB xenograft mouse model. This mouse model recapitulate the highly aggressive and invasive growth pattern of human NB [24]. NGP-luciferase cell xenografts were generated, imaged, randomized into two groups, and treated with either OTSSP167 (10 mg/kg/d) or DMSO (vehicle control) daily for 14 days. Subsequently, tumors were harvested, photographed, and weighted. Results showed that OTSSP167 significantly inhibit *in vivo* NB tumor growth in comparison to controls (Figure 6A, 6B). We then tested the effect of OTSSP167 on Rb phosphorylation in NB *in vivo*. In this experiment, we treated mice bearing the established tumors with either OTSSP167 (10 mg/kg/d) or DMSO (vehicle control) daily for 48 hours. Subsequently, tumors were harvested and analyzed. We found that MELK and p-Rb levels were significantly inhibited and PARP cleavage levels were increased in tumors from the OTPSS167 treated mice in comparison to tumors from vehicle treated mice (Figure 6C). These *in vivo* results further confirmed our *in vitro* observations that inhibion of MELK inhibit the Rb protein function to block cell cycle progression and induce apoptosis in NB. Based on these data, we suggest a working model for MELK function in NB cells (Figure 6D). Our results demonstrate the potential of MELK inhibition by OTSSP167 as a novel therapeutic approach for high-risk NB.

## DISCUSSION

Several transcription factors, such as oncoprotein FoxM1 and c-JUN, have been reported to promote MELK in cancer cells [25, 26]. However, in NB, the mechanism of MELK regulation still remains unclear. The gene expression analysis of NB tumors from patients revealed that *MELK* strongly correlates with *MYCN* expression and amplification. We therefore hypothesize that MYCN might promote *MELK* expression in NB. Our results demonstrate that *MELK* is a direct transcription target of MYCN/MYC in NB cells. Genetic and small molecule-mediated MELK inhibition was able to significantly suppress cell proliferation and induce apoptosis. MELK inhibition is a promising therapeutic strategy for high-risk NB patients.

Some studies have reported that *MELK* mutagenization mediated by CRISPR/Cas9 had no effects on basal breast cancer cell lines or cell lines from six other cancer types [27]. In contrast, our results showed that MELK knockdown using sh-RNA in four different NB cell lines dramatically inhibited cell viability and colony formation, a key indicator of the crucial role of MELK on cell survival in NB cells. It is likely that MELK is required for cell proliferation in NB but is not essential in some of other cancer types. Further study is needed to clarify the role of MELK in different cancer types.

MELK inhibitor OTSSP167 with a potential off-target effect should be considered. Studies have reported that MELK-knockout cells remain sensitive to OTSSP167 since OTSSP167 blocks cell proliferation through an off-target mechanism [27]. In addition, OTSSP167 is reported to show off-target activity against Aurora B, BUB1, and Haspin kinases in HeLa and MCF7 cells [28]. According to Kinome scan data, OTSSP167 is in fact a non-selective kinase inhibitor (http://lincs.hms.harvard.edu/db/sm/10337-102). Although related off-target effects are yet to be elucidated, OTSSP167 is still capable in directly reducing MELK levels and inducing apoptosis in NB cell lines tested.

Another confounding issue is to determine which signaling pathways regulate MELK kinase activity. MELK kinase mediates a large variety of cell characteristics including but not limited to cell cycle control, cell proliferation, apoptosis, cell migration, oncogenesis, cancer resistance, and recurrence [10]. Further, MELK functions through multifaceted signaling involving proteins such as p53 [29], Mcl-1 [30], Rb [31], CDK1, CDK25B, MAPK [32, 33]. In our study, we found that MELK kinase inhibition in NB showed inhibitory effect on Rb phosphorylation and cell proliferation. Signaling pathway that modulates MELK activity in NB cells should be further investigated.

In summary, our data showed that MELK is a transcriptional target of MYCN/MYC and plays an oncogenic role in NB. Inhibition of MELK, inhibit NB growth by blocking the cell cycle progression due to inhibition of Rb functions. The small molecule MELK inhibitor, OTSSP167 potently inhibit NB tumor growth, and reprents a promising therapeutic strategy for high-risk NB.

## MATERIALS AND METHODS

### Clinical patient cohorts

The Kocak neuroblastoma patient dataset (N = 649) and tumor neuroblastoma dataset SEGC (N = 498) that includes microarray profiles of unique primary tumors are publically available in the R2: Genomic Analysis and Visualization Platform database (http://hgserver1.amc.nl/cgibin/r2/main.cgi). This platform also support the multi-parametric analysis of NB patient outcome with gene expression.

### Cell culture and reagents

NB cell lines used in this study were routinely cultured and maintained as described previously [18, 19]. Cell lines were verified via genotyping within the past 6 months and were tested for Mycoplasma contaminations on a monthly basis. Anti-MELK (A303-136A) was purchased from Bethyl Laboratories. Anti-PARP (9532), anti-Caspase 3 (9662), anti-p-Rb (9308), anti-Rb (9309), anti-Mouse (7076), and anti-Rabbit (7074) antibodies were purchased from Cell Signaling Technology. Anti-MYCN (sc-56729) and anti-MYC (sc-40) were from Santa Cruz Biotechnology. Anti-β-actin (A2228) primary antibody was obtained from Sigma-Aldrich Corp (St. Louis, MO, USA). OTSSP167 (HY-15512) was purchased from Medchem Express (Monmouth Junction, NJ, USA).

### Immunoblotting assay

Immunoblotting assays were performed as described previously [34]. Briefly, cells were lysed using RIPA buffer and cell lysates were collected after centrifuging for 15 minutes at 13,000 rpm. Protein concentrations were determined using Bradford assay following the manufacturer's instructions (Bio-Rad). Protein samples were separated by SDS-PAGE, transferred to PVDF membranes (Bio-Rad), blocked with 5% milk, and probed with indicated primary antibodies overnight at 4°C. The membranes were then incubated with anti-mouse or anti-rabbit IgG conjugated with horseradish peroxidase for 1 hour and blots were developed using ECL-Plus Western detection system (GE HealthCare) for visualization.

### Cell cycle analysis

Cells were treated with indicated concentrations of OTSSP167 and washed with ice-cold PBS. Cells were then harvested and centrifuged for 5 min at 500 X g and 4°C. Solutions were then fixed with 3 mL 70% ice cold methanol. The fixed cells were stained with 50 μg/mL Propidium iodide (PI) solution and were analyzed by flow cytometry (FCS Express 4 Software). The data showed the distribution of the three cell cycle phases (G1, S, G2/M).

### Cell proliferation and soft agar colony formation assay

Cell proliferation was measured using Cell Counting Kit-8 (Dojindo Laboratories) in accordance to the manufacturer's instructions. NB cells were seeded at 5000 cells/well in a 96-well microtiter plate and after 24 hours of incubation, were treated with increasing concentrations of OTSSP167. Cellular proliferation was measured 72 hours post-incubation by adding CCK-8 reagent and was monitored for optical density at 450 nm using a microplate reader. Each experiment was performed in replicates of six and background reading of the media was subtracted from each well for result standardization. Soft agar colony formation assays were performed using standard conditions as described previously [24]. All assays were performed in triplicates and repeated three times with proper controls.

### Generation of gene knockdown and overexpression NB cell lines

Trc2 lentiviral shRNA vectors (Sigma-Aldrich Inc.) were used to generate *MYC*, *MYCN* and *MELK* knockdown cell lines. The following were shRNA sequences used: *MYCN*-sh-1: 5’- AATTCTTACACTGCCTGTATA-3’, *MYCN*-sh-2: 5’-AATCTCTGTTATGTACTGTAC-3’. *MYC*-sh-1: AATGTCCTGAGCAATCACCTA, *MYC*-sh-2: AATGTCCTGAGCAATCACCTA. *MELK*-sh-1: 5’-AAGTTCATTGGAACTACCAAC-3’, *MELK*-sh-2: 5’-AATTGATGGATTCTTCCATCC-3’. The lenti-viral expression vectors encoding human MYCN and MYC open reading frame (ORF) were used to generate *MYC* and *MYCN* overexpression NB cell lines. These vectors were used to generate virus and transduce NB cell lines as described previously [34].

### Quantitative reverse transcription-PCR

Gene transcription levels were measured using the qRT-PCR method as described previously [24]. Total RNA was extracted using TRIzol LS Reagent (Invitrogen) and the concentration was measured. Quantitative PCR was performed in triplicate using SensiFAST SYBR Hi-ROX One-Step Kit according to the manufacturer's instructions (Bio-73005, Bioline). The mRNA level for each gene was detected by Applied Biosystems™ Real-Time PCR Instruments. Primers used in this study were *MYCN*: Forward 5’-AGAGGACACCCTGAGCGATTC-3’, Reverse 5’-CATAGTTGTGCTGCTGGTGGA-3’; *MYC*: Forward 5’-CTCCATGAGGAGACACCGCCCA-3’, Reverse 5’-AAGGTGATCCAGACTCTGACCT-3’; *MELK*: Forward 5’-ATAGCTACCATCTCTCCAGTA-3’ and Reverse 5’-CTTGCAAGAGGACTATGAAAG-3’, respectively.

### ChIP-qPCR assay

ChIP-qPCR assays were performed using the ChIP-IT Express Kit (Active Motif) in accordance to the manufacturer's instructions as described previously [19]. The antibodies used for chromatin immune-precipitation were anti-MYCN (OP13, Calbiochem Inc.) and control mouse IgG (12-371, EMD Millipore). The ChIP-purified DNA was analyzed by qPCR. ChIP-MELK primers were designed using our previously published MYCN ChIP-seq dataset which used an MYCN3 cell line under both MYCN amplified and non-amplified conditions [18]. ChIP-MELK primers were designed using the UCSC genome browser and Primer3 software (www.SimGene.com). ChIP-MELK primers were Forward- 5’-GAGAACTGTGACTGCCAGAGG-3’ and Reverse- 5’-TGTGGAGCCGTGAAAGGGAT-3’. ChIP-negative control primers were Forward- 5’- ATGGTTGCCACTGGGGATCT -3’ and Reverse- 5’- TGCCAAAGCCTAGGGGAAGA -3’.

### *In vivo* xenograft assays

Four to six week-old female inbred athymic immunodeficient Nude mice (Nu/Nu) were purchased from Taconic Biosciences and used for all xenograft studies. Mice were implanted using a previously described orthotopic xenograft model [24, 34]. All mice were handled according to protocols approved by the Institutional Animal Care and Use Committee of Baylor College of Medicine. In the mice, 1 × 10^6^ NGP NB cells were surgically implanted in the sub-renal capsule through a transverse incision over the left flank. Tumor growth was monitored twice a week with bioluminescent imaging (IVIS Lumina XR System, Caliper Life Sciences). Mice bearing similar sized tumors were randomly divided into groups and were treated with either DMSO or OTSSP167 (10 mg/kg, intraperitoneal injection once daily) for 14 days. After treatment, tumors were harvested. Tumors were either weighted and photographed for analysis or lysed for immunoblotting.

### Statistical analysis

All values were presented as the mean ± standard deviation (SD). A two-tailed Student's t-test and ANVOA were used to determine the statistical significance among drug treatment groups. Each assay was repeated at least twice, and representative results were presented. *P* < 0.05 was considered to be statistically significant. The IC_50_ value was calculated with Graphpad Prism 5 software (La Jolla, CA). Survival analyses were performed using Kaplan-Meier method and two-sided log-rank tests.
